# Characteristics of Hospitals Participating in the Transforming Episode Accountability Model

**DOI:** 10.1001/jamahealthforum.2025.1996

**Published:** 2025-07-11

**Authors:** Sukruth A. Shashikumar, Jie Zheng, Thomas C. Tsai, E. John Orav, Arnold M. Epstein, Karen E. Joynt Maddox

**Affiliations:** 1 Department of Medicine, Brigham and Women’s Hospital, Boston, Massachusetts; 2 Harvard Medical School, Boston, Massachusetts; 3 Department of Health Policy and Management, Harvard T.H. Chan School of Public Health, Boston, Massachusetts; 4 Department of Surgery, Brigham and Women’s Hospital, Boston, Massachusetts; 5 Department of Biostatistics, Harvard T.H. Chan School of Public Health, Boston, Massachusetts; 6 Cardiovascular Division, Department of Medicine, Washington University School of Medicine, St Louis, Missouri; 7 Center for Advancing Health Services, Policy & Economics Research, Washington University, St Louis, Missouri

## Abstract

This cross-sectional study compares the hospital-, market-, patient-, and spending-level characteristics of hospitals mandated vs not mandated to participate in the Transforming Episode Accountability Model (TEAM) bundled payment program.

## Introduction

The Centers for Medicare & Medicaid Services (CMS) recently announced the Transforming Episode Accountability Model (TEAM), a mandatory bundled payment program. Under TEAM, hospitals in selected US regions are accountable for spending on episodes of care, spanning admission to 30 days after discharge, for 5 surgical procedures.^1^ If hospitals meet spending targets, they receive a financial bonus from CMS; if not, they must pay CMS a penalty.

Prior bundled payment models were voluntary, allowing hospitals to start and end participation at will. This voluntary system was associated with selective enrollment by hospitals with high operating margins and low shares of marginalized patients.^2^ The selection bias, in turn, preceded payout of bonuses that exceeded spending reductions under the model, precipitating net financial losses for CMS.^3^

Moving from voluntary to mandatory participation addresses selection bias and increases participation from safety-net hospitals, but it has its own challenges. Safety-net hospitals mandated to participate in the Comprehensive Care for Joint Replacement (CJR) bundled payment model reduced spending on a magnitude similar to that for other hospitals yet were disproportionately penalized because they had lower (harder-to-meet) spending targets.^1^ Because TEAM sets spending targets similar to those of CJR, there is concern that safety-net hospitals might also disproportionately perform poorly under TEAM. Therefore, we sought to understand the characteristics of hospitals mandated to participate in TEAM as CMS looks to address selection bias and more equitably distribute financial incentives under bundled payment models.

## Methods

We compared characteristics of hospitals selected vs not selected for TEAM using χ^2^ and *t* tests. We also compared spending between these groups after adjusting for patient- and hospital-level characteristics (eMethods in Supplement 1). The Human Research Protection Office at Washington University approved this cross-sectional study and waived informed consent because it was not human participant research. We followed the STROBE reporting guideline.

We estimated episode spending for TEAM’s 5 inpatient bundles: coronary artery bypass graft (CABG), lower extremity joint replacement (LEJR), major bowel procedure, spinal fusion, and surgical hip and femur fracture treatment (SHFFT). Analysis was conducted between November 2024 and March 2025 using SAS software (SAS Institute).

## Results

We identified 716 hospitals participating in TEAM (Table 1). Compared with nonparticipants, participants were larger (263 vs 230 beds) and more often not-for-profit (506 [70.7%] vs 1336 [65.3%]), teaching (405 [56.6%] vs 1030 [50.3%]), and safety-net (99 [13.8%] vs 177 [8.7%]) hospitals. Participants were in wealthier counties (mean [SD] household income, $76 023 [$23 830] vs $67 201 [$15 487]) and served a higher share of patients with dual Medicare-Medicaid enrollment (25.9% vs 23.3%).

**Table 1.** Characteristics of Hospitals Participating and Not Participating in TEAM^a^

Characteristic	TEAM hospitals^b^	Nonparticipant hospitals	*P* value^c^
Hospital level			
Total No. (%)	716 (100)	2046 (100)	NA
Beds, mean (SD) No.	263.0 (253.6)	230.0 (230.3)	.001
Urban location, No. (%)	684 (95.5)	1994 (97.5)	<.001
Ownership, No. (%)			
For-profit	126 (17.6)	482 (23.6)	<.001
Not-for-profit	506 (70.7)	1336 (65.3)	.009
Public	84 (11.7)	228 (11.1)	.67
Geography, No. (%)			
Northeast	211 (29.5)	217 (10.6)	<.001
Midwest	90 (12.6)	574 (28.1)	<.001
South	234 (32.7)	857 (41.9)	<.001
West	181 (25.3)	398 (19.5)	.001
Teaching hospital (major or minor), No. (%)	405 (56.6)	1030 (50.3)	.004
Member of a system, No. (%)	559 (78.1)	1586 (77.5)	.76
Safety-net hospital, No. (%)	99 (13.8)	177 (8.7)	<.001
DSH percentage points, mean (SD)	32.9 (18.7)	29.2 (16.9)	<.001
Hospital total margin, mean (SD)	0.01 (0.13)	0.02 (0.18)	.12
Hospital operating margin, mean (SD)	0.00 (0.15)	0.01 (0.22)	.07
County level			
Census population in 2020, mean (SD) No.	694 185 (752 568)	1 071 448 (2 027 134)	<.001
Household income in 2021, mean (SD) of the median, $	76 023 (23 830)	67 201 (15 487)	<.001
Medicare Advantage penetration in 2022, mean (SD) %	45.6 (12.8)	46.5 (12.2)	.10
SNF total beds in 2022, mean (SD) No.	2754 (2753.8)	4399 (8291.6)	<.001
Rehabilitation hospitals in 2021, mean (SD) No.	1.01 (1.24)	2.50 (4.36)	<.001
Hospital market share, mean (SD) proportion	0.42 (0.38)	0.44 (0.40)	.10
Patient level			
Dual Medicare-Medicaid enrollment, %	25.9 16.7)	23.3 (17.2)	<.001
Black race, mean (SD) %^d^	8.6 (12.3)	8.0 (12.1)	.26
Hispanic ethnicity, mean (SD) %^d^	6.4 (9.7)	5.8 (11.6)	.88
Annual discharges, mean (SD) No.	2162.0 (2408.47)	1894.3 (2033.73)	.004
Inpatient bundles			
CABG, mean (SD)	13.4 (28.6)	13.0 (25.9)	.78
LEJR, mean (SD)	59.1 (144.54)	43.9 (61.92)	<.001
Major bowel procedure, mean (SD)	27.7 (38.6)	25.6 (33.7)	.17
Spinal fusion, mean (SD)	8.5 (16.3)	7.7 (13.4)	.17
SHFFT, mean (SD)	36.1 (38.1)	32.5 (35.7)	.02

Abbreviations: CABG, coronary artery bypass graft; DSH, disproportionate share hospital; LEJR, lower extremity joint replacement; NA, not applicable; SHFFT, surgical hip and femur fracture treatments; SNF, skilled nursing facility; TEAM, Transforming Episode Accountability Model.

^a^ Data sources include the 2022 American Hospital Association survey, 2023 Area Health Resources File, and 2023 Medicare enrollment and inpatient claims data.

^b^ Twenty-five hospitals participating in TEAM could not be linked to characteristics and were excluded from analyses.

^c^ Two-tailed *P* < .05 was considered statistically significant.

^d^ Race and ethnicity data were collected because (1) prior research has demonstrated that hospitals serving patients from racial and ethnic minority groups disproportionately face financial penalties under many value-based payment programs, and (2) voluntary participation under prior bundled-payment programs has been associated with selective enrollment by hospitals with low shares of marginalized patients; thus, it is important to monitor the racial makeup of patients at hospitals participating and not participating in TEAM. Black race and Hispanic ethnicity are the only groups reported due to the low number of patients in other minority groups and due to space constraints.

Participants and nonparticipants had similar episode volume for most bundles. Spending ranged from $28 315 for LEJR to $49 551 for CABG. For all bundles, 30-day episode spending was higher among participants than nonparticipants (Table 2). Among participants, postacute care (PAC) spending varied from $5457 for CABG to $17 340 for SHFFT, accounting for 11.0% and 45.3%, respectively, of 30-day spending.

**Table 2.** Overall and Subcomponents of Adjusted 30-Day Episode Spending at Hospitals Participating and Not Participating in TEAM

Spending	Hospitals, $		*P* value^a^
	TEAM participants	Nonparticipants	
Overall 30-d episode			
CABG	49 551	46 968	<.001
LEJR	28 315	25 914	<.001
Major bowel procedure	31 820	29 326	<.001
Spinal fusion	34 092	31 587	<.001
SHFFT	38 271	35 447	<.001
30-d PAC^b^			
CABG	5457	5486	.68
LEJR	9397	8813	<.001
Major bowel procedure	4368	4308	.23
Spinal fusion	6763	6416	.005
SHFFT	17 340	16 269	<.001
30-d SNF			
CABG	2090	1718	<.001
LEJR	5708	5068	<.001
Major bowel procedure	2756	2368	<.001
Spinal fusion	2596	2075	<.001
SHFFT	11 951	10 157	<.001
30-d IRF			
CABG	2574	3020	<.001
LEJR	2638	2996	<.001
Major bowel procedure	1164	1557	<.001
Spinal fusion	3834	3987	.19
SHFFT	5124	5869	<.001
30-d HHA			
CABG	793	749	<.001
LEJR	1051	749	<.001
Major bowel procedure	449	384	<.001
Spinal fusion	334	354	.03
SHFFT	265	243	<.001

Abbreviations: CABG, coronary artery bypass graft; HHA, home health agency; IRF, inpatient rehabilitation facility; LEJR, lower extremity joint replacement; PAC, postacute care; SHFFT, surgical hip and femur fracture treatments; SNF, skilled nursing facility; TEAM, Transforming Episode Accountability Model.

^a^ Two-tailed *P* < .05 was considered statistically significant.

^b^ Postacute care spending includes spending for SNFs, IRFs, and HHAs.

## Discussion

Hospitals mandated to participate in TEAM were larger, served more marginalized patients, and were often part of the safety net. This finding supports concerns that a large share of hospitals could bear disproportionate penalties under TEAM, as under CJR.^1,4^

Generally, TEAM participants had higher spending than nonparticipants, suggesting potential to reduce spending. However, this potential might vary across bundles. Participants in bundled payment generally lower overall episode spending by reducing PAC spending.^5,6^ Greater decreases might thus be expected in procedures for which PAC represents a greater portion of episode spending, such as LEJR and SHFFT bundles.^1,6^

Study limitations include the exclusion of 25 hospital participants that could not be linked to characteristics. We estimated spending for 5 inpatient bundles because they encompass most episodes under TEAM; TEAM also includes 2 outpatient bundles. Results of this study offer insight into the structural and spending characteristics of TEAM participants, including implications for equity and financial success under the model.

## Notes

Supplement 1.
eMethods.

Supplement 2.
Data Sharing Statement

## References

[1] Shashikumar SA, Ryan AM, Joynt Maddox KE. Medicare’s new Mandatory Bundled-Payment Program - are we ready for TEAM? N Engl J Med. 2024;391(22):2065-2067. doi:10.1056/NEJMp2410850 39620465

[2] Joynt Maddox KE, Orav EJ, Zheng J, Epstein AM. Characteristics of hospitals that did and did not join the Bundled Payments for Care Improvement–Advanced program. JAMA. 2019;322(4):362-364. doi:10.1001/jama.2019.7992 31334781 PMC6652152

[3] Shashikumar SA, Gulseren B, Berlin NL, Hollingsworth JM, Joynt Maddox KE, Ryan AM. Association of hospital participation in Bundled Payments for Care Improvement Advanced with Medicare spending and hospital incentive payments. JAMA. 2022;328(16):1616-1623. doi:10.1001/jama.2022.18529 36282256 PMC9597389

[4] Shashikumar SA, Ryan AM, Joynt Maddox KE. Equity implications of hospital penalties during 4 years of the Comprehensive Care for Joint Replacement Model, 2016 to 2019. JAMA Health Forum. 2022;3(12):e224455. doi:10.1001/jamahealthforum.2022.4455 36459162 PMC9719045

[5] Agarwal R, Liao JM, Gupta A, Navathe AS. The impact of bundled payment on health care spending, utilization, and quality: a systematic review. Health Aff (Millwood). 2020;39(1):50-57. doi:10.1377/hlthaff.2019.00784 31905061

[6] Shashikumar SA, Zheng J, Orav EJ, Epstein AM, Joynt Maddox KE. Changes in cardiovascular spending, care utilization, and clinical outcomes associated with participation in Bundled Payments for Care Improvement–Advanced. Circulation. 2023;148(14):1074-1083. doi:10.1161/CIRCULATIONAHA.123.065109 37681315 PMC10540757

