# Supplementary material for: Characteristics of Hospitals Participating in the Transforming Episode Accountability Model
Source: JAMA Health Forum. 2025 Jul 11;6(7):e251996. doi: 10.1001/jamahealthforum.2025.1996 (PMC12254884; doi:10.1001/jamahealthforum.2025.1996)
Supplement: Supplement 1. — eMethods. [file jamahealthforum-e251996-s001.pdf]

Supplemental Online Content

Shashikumar SA, Zheng J, Tsai TC, Orav EJ, Epstein AM, Joynt Maddox KE. Characteristics of hospitals participating in the Transforming Episode Accountability Model. *JAMA Health Forum*. 2025;8(7):e251996. doi:10.1001/jamahealthforum.2025.1996

eMethods

This supplemental material has been provided by the authors to give readers additional information about their work.

© 2025 Shashikumar SA et al. *JAMA Health Forum*

eMethods

Model description and participant selection

The five procedures in the Transforming Episode Accountability Model (TEAM), which will launch in 2026, are coronary artery bypass grafts (CABGs), lower extremity joint replacements (LEJRs), major bowel procedures, spinal fusions, and surgical hip/femur fracture treatment (SHFFTs).

Per the Centers for Medicare & Medicaid Services (CMS) Final Rule 89 FR 68986, TEAM participants are defined as acute care hospitals paid under the Inpatient Prospective Payment System (IPPS) with a CMS Certification Number primary address located in one of the geographic areas selected for participation in TEAM. All acute care hospitals in Maryland are excluded from being TEAM participants because these hospitals are not currently paid under the IPPS.

CMS chose hospitals to participate in TEAM based on their location in selected Core-Based Statistical Areas (CBSAs). CBSAs include a core area with a substantial portion of the population in adjacent communities having a high degree of economic and social integration with that core. CBSAs located entirely in the state of Maryland, and certain CBSAs that straddle Maryland and another state, were not eligible for selection into TEAM; also ineligible were CBSAs in which no episodes were initiated at hospitals for any of the five procedures included in TEAM between January 1, 2022 and June 30, 2023 (89 FR 68986 §X.A.4.b). After applying these criteria, 803 CBSAs were available for selection into TEAM.

© 2025 Shashikumar SA et al. *JAMA Health Forum*

Instead of taking a simple random sampling where all eligible CBSAs had the same chance for selection into the model, CMS first grouped CBSAs into 18 strata based on average historical episode spending, number of hospitals, number of safety-net hospitals, and the CBSA's exposure to prior CMS bundled payment models. CBSAs with low past exposure to bundled payment models and those with a high number of safety-net hospitals were oversampled (89 FR 68986 §X.A.4.d). The selection probability for a given CBSA differed across strata, but all CBSAs within a particular stratum had the same chance of being selected. CMS then executed a stratified random sampling such that 188 CBSAs (~25% of eligible CBSAs) were selected to participate in TEAM. All hospitals in selected CBSAs are required to participate in the model.

CMS has made publicly available the list of hospitals selected for TEAM. Therefore, to identify TEAM nonparticipants, we identified the 803 CBSAs initially eligible for selection into TEAM, and, of those, the 188 CBSAs ultimately selected, through publicly available lists in CMS's Final Rule 89 FR 68986. We categorized the hospitals in initially eligible, but ultimately non-selected, CBSAs as "TEAM nonparticipants."

Data sources

We compared hospital, market, patient, and spending characteristics using the 2022 American Hospital Association survey, 2023 Area Health Resources File, and 2023 Medicare enrollment and inpatient claims data.

Statistical analyses

Following CMS's finalized TEAM methodology, we estimated multivariable regression models to compare spending patterns between model participants and nonparticipants. The full methods

© 2025 Shashikumar SA et al. *JAMA Health Forum*

are detailed in 89 FR 68986 §X.A.5.d.(4). In brief, CMS will risk-adjust spending in TEAM by a variety of patient- and hospital-level variables, which differ for each procedure in the program, as detailed below.

We followed CMS methodology and adjusted spending for all 5 TEAM bundles for the following 6 variables: patient age bracket, Hierarchical Condition Category (HCC) count, prior post-acute care use, and social risk, as well as hospital size and safety-net status. The exception is the SHFFT bundle, which per CMS methodology was not adjusted for prior post-acute care use. The patient age bracket variable includes 4 categories (<65 years, 65 to < 75 years, 75 to < 85 years, and 85+ years). The HCC count variable segments patients into 5 categories, based on having 0, 1, 2, 3, or 4+ assigned HCC conditions in a 90-day lookback period. The prior post-acute care use variable flags whether the patient has had an encounter with a long-term care facility, skilled nursing facility, home health agency, or inpatient rehabilitation facility. The social risk variable segments patients into 2 categories: those living in >8th decile for state Area Deprivation Index (ADI), living in >80th percentile for national ADI, eligible for the low-income subsidy, or eligibility for full Medicaid benefits; or those that do not meet any of those criteria. The hospital size variable includes 4 categories (≤250 beds, 251-500, 501-850, or 850+ beds). To be categorized as a safety-net hospital, hospitals must either (1) exceed the 75th percentile of the proportion of Medicare beneficiaries considered dually eligible for Medicare and Medicaid across all Prospective Payment System acute care hospitals, or (2) exceed the 75th percentile of the proportion of Medicare beneficiaries partially or fully eligible to receive Part D low-income subsidies across all Prospective Payment System acute care hospitals. We were unable to incorporate definitions that included eligibility for the Part D low-income subsidy, as we did not have access to those

© 2025 Shashikumar SA et al. *JAMA Health Forum*

data. Lastly, for spending in the LEJR bundle, CMS also adjusts for disability as the original reason for Medicare enrollment, having dementia without complications, and procedure-related variable (ankle procedure or reattachment, partial hip procedure, partial knee arthroplasty, total hip arthroplasty or hip resurfacing procedure, and total knee arthroplasty), which we did as well.

In addition to the variables above, CMS adjusts spending for each bundle with specific HCCs, which differ for each bundle. Thus, following CMS methodology, we adjusted spending for CABGs for HCCs 18, 46, 58, 84, 85, 86, 96, 103, 111, 112, and 134; LEJRs for HCCs 8, 18, 22, 58, 78, 85, 86, 103, 111, 112, 134, and 170; major bowel procedures for HCCs 11, 18, 21, 33, 82, 85, 86, 103, 111, 112, 134, and 188; spinal fusions for HCCs 8, 18, 22, 40, 58, 85, 86, 96, 103, 111, 112, and 134; and SHFFTs for HCCs 18, 22, 82, 83, 84, 85, 86, 96, 103, 111, 112, 134, 157, 158, 161, and 170.

All analyses were conducted using SAS (SAS Institute). 2-tailed $p < 0.05$ was considered significant. The Human Research Protection Office at Washington University approved this study.

© 2025 Shashikumar SA et al. *JAMA Health Forum*
