# Supplementary material for: Characteristics of Hospitals Participating in the Transforming Episode Accountability Model
Source: JAMA Health Forum. 2025 Jul 11;6(7):e251996. doi: 10.1001/jamahealthforum.2025.1996 (PMC12254884; doi:10.1001/jamahealthforum.2025.1996)
Supplement: Supplement 2. — Data Sharing Statement [file jamahealthforum-e251996-s002.pdf]

Data Sharing Statement

Shashikumar. Characteristics of Hospitals Participating in the Transforming Episode Accountability Model. *JAMA Health Forum*. Published July 11, 2025. doi:10.1001/jamahealthforum.2025.1996

Data
Data available: Yes
Data types: Data (not involving human participants)
How to access data: Data on program participation are public; we are glad to share the data or interested researchers can download from https://www.cms.gov/priorities/innovation/innovation-models/team-model The patient-level data are accessed through a specific data use agreement on Medicare's Virtual Research Data Center and cannot be shared.
When available: With publication

Supporting Documents
Document types: None

Additional Information
Who can access the data: Data are public
Types of analyses: Data are public
Mechanisms of data availability: Data are public
Any additional restrictions: Participation data are public. Patient-level data cannot be shared (and in fact is only accessed virtually and cannot be downloaded).
